# In vivo clearance of surfactant lipids during acute pulmonary inflammation.

**DOI:** 10.1186/1465-9921-5-8

**Published:** 2004-07-23

**Authors:** Jaret L Malloy, Jo Rae Wright

**Affiliations:** 1 Address: Box 3709, Department of Cell Biology, Duke University Medical Center, Durham, NC 27710, USA

## Abstract

**Background:**

A decrease in pulmonary surfactant has been suggested to contribute to the lung dysfunction associated with pulmonary inflammation. A number of studies have implicated surfactant clearance as a possible mechanism for altered pool sizes. The objective of the current study was to specifically investigate the mechanisms of surfactant clearance in a rodent model of acute pulmonary inflammation.

**Methods:**

Inflammation was induced by intrapulmonary instillation of lipopolysaccharide (LPS: 100 μg/kg). Lipid clearance was assessed at 18 and 72 hours post-LPS instillation by intratracheal administration of radiolabel surfactant-like liposomes 2 hours prior to isolation and analysis of inflammatory cells and type II cells.

**Results:**

At both 18 and 72 hours after LPS instillation there was significantly less radioactivity recovered in the lavage fluid compared to respective control groups (p < 0.05). At both time points, the number of cells recovered by lavage and their associated radioactivity was greater compared to control groups (p < 0.01). There was no difference in recovery of radioactivity by isolated type II cells or other cells obtained from enzymatic digestion of lung tissue.

**Conclusion:**

These results show that increased clearance of surfactant lipids in our model of acute pulmonary inflammation is primarily due to the inflammatory cells recruited to the airspace and not increased uptake by alveolar type II cells.

## Background

Pulmonary surfactant is a phospholipid-protein complex that lines the inner surface of the lung and is essential for normal pulmonary function. Surfactant acts to promote lung stability by reducing surface tension within the lung, while also protecting against inhaled pathogens. Surfactant is composed of approximately 90% lipids and 10% proteins by weight. The lipid component is primarily phospholipids with phosphatidylcholine (PC) being the most abundant, and the protein component comprised of four surfactant-associated proteins designated SP-A, SP-B, SP-C and SP-D [1]. The reduction of surface tension within the lung is a result of the interaction between surfactant phospholipids and the two hydrophobic surfactant proteins, SP-B and SP-C [1], while the two hydrophilic proteins, SP-A and SP-D, are members of a family of innate immune molecules called collectins [2]. Collectins opsonize bacteria and viruses and enhance their phagocytosis by macrophages and neutrophils [2].

Alterations of the pulmonary surfactant system, including decreased total surfactant levels, have been implicated in the pathophysiology of acute lung injury. Multiple studies of patients with a variety of lung diseases have shown that surfactant levels are decreased in the inflamed, injured, or infected lung [3-5]. In agreement, decreases in alveolar surfactant lipid pools have also been observed in several animal models of lung inflammation induced by both direct insults to the lung, such as bacterial infection [6], oxygen toxicity [7,8], endotoxin administration [9-11], and by indirect insults, such as N-nitroso-N-methylurethane [12] and cecal ligation and perforation [13].

Alveolar metabolism of surfactant is a complex process, primarily involving type II epithelial cells that synthesize, secrete and clear surfactant from the airspaces [14], along with phagocytic cells such as macrophages and neutrophils that participate in surfactant clearance [15,16]. In a situation of pulmonary inflammation, altered type II cell metabolism has been thought to play a role in the alterations of surfactant lipid levels. Viviano et al. observed a decrease in alveolar surfactant levels and a corresponding increase in intracellular surfactant after lipopolysaccharide (LPS) administration [10]. Additionally, exposure of rat lungs ventilated *ex vivo *to LPS resulted in the presence of giant lamellar bodies within the type II cells [17,18]. Collectively these studies suggest that type II cell metabolism is altered after LPS administration and that a possible explanation for decreased surfactant pool size may be increased clearance of surfactant lipids by the type II cells. Additional experimental evidence has also implicated recruited inflammatory cells as having an impact on surfactant pool sizes. Both neutrophils and macrophages recovered from LPS exposed lungs had a greater capacity to internalize surfactant-like lipids compared to control cells *in vitro *[16]. Therefore these recruited inflammatory cells may also have a significant impact on surfactant pool size by increasing the overall surfactant lipid clearance.

The objective of the current study was specifically to investigate the mechanisms of *in vivo *surfactant clearance in a rodent model of acute pulmonary inflammation induced by intrapulmonary instillation of LPS. We hypothesized that during pulmonary inflammation there is altered clearance of alveolar phospholipid by both alveolar type II cells and inflammatory cells within the airspaces. We analyzed *in vivo *clearance of surfactant lipids by a variety of pulmonary cells at 18 and 72 hours after LPS instillation. Results indicated that increased clearance of surfactant lipids in our model of acute pulmonary inflammation is primarily due to enhanced lipid uptake by the inflammatory cells recruited to the airspace, whereas uptake by type II cells was unaltered. However the *in vivo *surfactant uptake by inflammatory cells in the current study was significantly less than that predicted by previous *in vitro *studies [16].

## Methods

### Materials

Dipalmitoylphosphatidylcholine (DPPC), egg phosphatidylcholine (PC), dipalmitoylphosphatidylglycerol (DPPG) and cholesterol were purchased from Avanti Polar Lipids (Birmingham, AL). L-α-dipalmitoyl [2-palmitoyl-9,10-^3^H(N)]PC was obtained from DuPont New England Nuclear (Boston, MA). Elastase for type II cell isolations was purchased from Worthington Biochemicals (Freehold, NJ). Dulbecco's PBS, DMEM, and fetal bovine serum (FBS) were obtained from Life Technologies (Gaithersburg, MD). Low-endotoxin BSA, O26:B6 *Escherichia coli *LPS, Rat IgG and all other chemicals were purchased from Sigma (St. Louis, MO). Chloroform and methanol were obtained from EM Science (Gibbstown, NJ).

### Preparation of Liposomes

Small unilamellar liposomes were prepared with a lipid composition similar to pulmonary surfactant: 52% DPPC, 26% egg PC, 15% DPPG and 7% cholesterol by weight with trace amounts of ^3^H-DPPC (12 μCi/mg phospholipid). The lipids were dried under nitrogen, reconstituted in 0.15 M saline and extruded from a French Press cell under 900 psi. This resulted in small unilamellar liposomes at a concentration of approximately 1 mg lipid/ml.

### Animal Model

Male pathogen-free Sprague Dawley rats (150–200 g; Taconic Farms, Germantown, NY) were used for the current study. For LPS animals, endotoxin (0.1 mg/kg of O26:B6 *E. coli *LPS) was suspended in 300 μl of sterile 0.15 M saline. Control animals received an equal volume (300 μl) of sterile 0.15 M saline. For instillation, animals were anesthetized with halothane such that they remained unconscious throughout the entire instillation procedure and had no cough reflex upon intubation. Animals were placed on a board at a 45° angle, intubated with an 18-gauge blunt ended catheter and either sterile saline or LPS suspension was instilled followed by five 1 ml boluses of air to facilitate the distribution of the instilled fluid. Two hours prior to killing, liposomes (100 μg lipid) were intratracheally instilled in all groups following the identical instillation procedure. Separate groups of both control and LPS animals were sacrificed at 18 or 72 hours after saline or LPS instillation.

### Whole Lung Studies

One control animal and one LPS animal were investigated simultaneously, thus all procedures were done in parallel. At 18 or 72 hours after saline or LPS instillation, animals were anesthetized with 0.3 mg of sodium pentobarbital and 700 U of heparin. After loss of toe pinch reflex, the trachea was cannulated and the rat was exsanguinated via transection of the descending aorta. The chest cavity was subsequently opened and the lungs perfused through the pulmonary artery with 40–50 ml of a calcium buffer (140 mM NaCl, 5 mM KCl, 2.5 mM Na_2_HPO_4_, 10 mM HEPES, 2.0 mM CaCl_2_, and 1.3 mM MgSO_4 _at 37°C). The lung from the first animal was carefully removed from the thoracic cavity and placed between saline soaked gauze pads while the second lung was perfused and removed in identical fashion. The time for lung perfusion and removal was approximately 10 minutes. After lungs were isolated from both a control and LPS animal, they were lavaged simultaneously with eight 10-ml volumes of EGTA buffer (140 mM NaCl, 5 mM KCl, 2.5 mM Na_2_HPO_4_, 10 mM HEPES, and 0.2 mM EGTA at 37°C). For each animal the individual lavages were collected and combined to make up the bronchoalveolar lavage fluid (BALF), which was immediately stored on ice. After the lavage procedure, individual lobes were dissected away from the major airways. The lung tissue was then cut into 5 mm pieces in 5 ml calcium buffer and subsequently homogenized with a Polytron PT-MR-2100. After complete homogenization, total lung tissue volume was diluted to 40 ml with calcium buffer and stored on ice. A small aliquot (2 ml) of the total BALF was removed and the remainder was centrifuged at 250 *g *for 10 minutes at 4°C to generate a pellet that was primarily BALF cells. The cell pellet was subsequently suspended in 10 ml of PBS. Lavage cell numbers were determined by a hemocytometer, viability determined by trypan blue exclusion and cell differential determined by Hemacolor staining of cytospins that were prepared using a Shandon Cytospin 2 centrifuge. Liposome-association in total BALF, cell-free BALF, BALF cells and homogenized lung tissue was determined by scintillation counting.

### Surfactant Phospholipid Measurement

Alveolar phospholipid levels were measured in the cell-free BALF by phospholipid-phosphorous measurement. Lipids were extracted using the method of Bligh & Dyer [19] and phospholipid levels were determined using a modification of the Duck-Chong phosphorous assay [20]. Briefly, 100 μl of 10% magnesium nitrate in methanol was added to the extracted lipids. After drying, the samples were ashed in a fume hood on an electric rack for approximately 1 min. After 1 ml of 1 M HCl was added, the samples were reheated on a heating block while covered for 15 min at 95°C. After cooling, a 66 μl aliquot of each sample was added to individual wells of a 96 well plate along with 134 μl of a dye consisting of 4.2% ammonium molybdate in 4.5 M HCl with 0.3% malachite green (1:3 vol/vol). The absorbency of each sample was read at 650 nm using a Biorad 550 microplate reader and compared to reference standards on the same plate.

### Surfactant Protein A (SP-A) Analysis

Relative quantities of alveolar SP-A from 18-hour control animals, 18-hour LPS animals and 72-hour LPS animals were determined by loading equal volumes of BALF on 15% SDS-PAGE gels under reducing conditions. Total BALF recovery was not different among the individual animals. Proteins were then transferred to nitrocellulose where SP-A was probed with a well characterized polyclonal rabbit anti-rat SP-A [21]. The nitrocellulose was subsequently developed using the ECL system (Amersham Pharmacia Biotech, Piscataway, NJ).

### Type II Cell Isolation

A separate cohort of animals was required for type II cell isolation studies. As with the whole lung studies, type II cell isolation was completed for one control and one LPS rat in parallel for both 18- and 72-hour time points after instillation of saline or LPS. The type II cell isolation procedure was described previously with minor modifications of the original protocol [22,23]. The modifications were designed for isolation of type II cells from inflamed lungs, and included increasing the elastase to 3,000 orcein units/lung, and increasing the surface area for IgG panning by two-fold. Briefly the isolation procedure involved killing the rats, removing the lungs and lavaging the lungs eight times as stated in *Whole Lung Studies. *Subsequently the lungs were lavaged twice with calcium buffer (37°C) and once with elastase solution (3000 orcein/40 ml calcium buffer; 37°C). The lungs were then filled with the elastase solution and suspended in warmed saline (37°C) for 20 minutes. Lung tissue was removed from the major airways, cut into 5-mm pieces and chopped 200 times with sharp scissors in 5 ml of the calcium solution along with 2 mg of DNase. The tissue suspension was added to a flask with 4 ml of FBS and 35 ml of calcium buffer and shaken vigorously for 2 min in a 37°C water bath. The resultant tissue suspension was strained through gauze, and then decreasing sizes of nylon mesh (150-, 15-, and 8-μm). The cell suspension was centrifuged at 250 *g *for 10 min at 4°C, the resulting cellular pellet was suspended in 20 ml of warm DMEM (37°C) and incubated on two IgG coated Petri dishes (150 × 15 mm) for 30 minutes at 37°C. After the incubation period, nonadherent cells were removed from the plate, washed once with PBS, suspended in calcium buffer and used for determination of radiolabel recovery associated with Type II cells. The remaining adherent cells were gently scraped off the plate in 5 ml of DMEM and transferred into a polypropylene tube; this population of cells is referred to as "plate cells" and consisted primarily of macrophages and/or neutrophils as described in detail below. The cells were washed once and suspended in calcium buffer for determination of associated radioactivity. A small aliquot from the isolated type II cells and plate cells was saved for differential cell count and viability. Cell purity was determined by counting a minimum of 250 cells from random fields after staining by the Papanicolaou method [22].

### Statistics

All data reported are means ± standard error (SE). Repetitions (n) used to calculate means ± SE were from independent experiments, not from replicates within an experiment. An analysis of variance (ANOVA) was used to determine differences between all experimental groups at a specific time point, followed by a Tukey post-hoc test for multiple comparisons. Significance was accepted when p < 0.05.

## Results

### LPS-induced alterations: BALF cells and alveolar surfactant

Table 1 reveals total alveolar cell numbers and differentials from the BALF of control and LPS groups killed at the 18- and 72-hour time points. Eighteen hours after instillation of LPS there were significantly more cells recovered in the BALF from the LPS treated animals compared to the saline control group (p < 0.01). Cell differentials from the two 18-hour groups revealed that the LPS group had primarily neutrophils in the BALF, whereas the cells recovered from the control group were predominantly macrophages. Seventy-two hours after LPS instillation there were significantly greater numbers of cells in the BALF of the LPS group compared to the 72-hour control group (p < 0.01), but significantly fewer numbers of cells compared to the LPS 18-hour group (p < 0.01). Cell differentials from the 72-hour groups revealed that the cells were predominately macrophages in both the LPS and control groups. Of note, there were no differences in cell numbers or differentials between the two control groups at the different time points.

**Table 1:** Bronchoalveolar lavage fluid characteristics from animals killed 18 and 72 hours after instillation of saline (Control) or lipopolysaccharide (LPS).

	Control 18 (n = 6)	LPS 18 (n = 6)	Control 72 (n = 6)	LPS 72 (n = 6)
Cell Numbers (10^6^)	12.3 ± 1.1	102.8 ± 8.1*	11.3 ± 0.9	57.3 ± 6.6*^#^
% Macrophages	98.2 ± 0.3	7.3 ± 0.8*	97.7 ± 0.4	90 ± 1.5*
% Neutrophils	1.8 ± 0.3	92.7 ± 0.8*	2.3 ± 0.4	10 ± 1.5*
Alveolar Phospholipid (mg PL/kg BW)	14.7 ± 0.7	12.8 ± 0.9	14.0 ± 0.4	12.9 ± 0.5

Abbreviations: PL = phospholipid; BW = body weight. Values are mean ± SEM. * = p < 0.01 vs. appropriate control; ^# ^= p < 0.01 vs. LPS 18.

**Figure 1:**
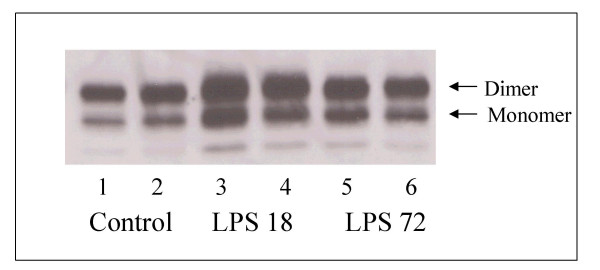
**Alveolar surfactant protein A (SP-A) levels. **Relative quantities of alveolar SP-A were measured by Western Blot. Equal volumes of bronchoalveolar lavage fluid (BALF) from the individual animals were utilized and total BALF recovery was not different among these animals. Lanes 1 and 2 represent individual animals that were lavaged 18 hours after saline instillation. Lanes 3 and 4 represent individual animals that were lavaged 18 hours after instillation of 100 μg/kg O26:B6 lipopolysaccharide (LPS). Lanes 5 and 6 represent animals lavaged 72 hours after instillation of 100 μg/kg 026:B6 LPS.

Table 1 also displays the total phospholipid levels measured in the BALF of the four experimental groups. There was an approximate 15% decrease in total phospholipid recovered by lung lavage from both LPS groups compared to their respective saline control groups, however this did not reach statistical significance. Of note, there was no difference in mean body weights among the four experimental groups (Con 18: 177 ± 7 g; LPS 18: 180 ± 10 g; Con 72: 186 ± 9 g; LPS 72: 192 ± 7 g). Figure 1 shows Western blot analysis of SP-A measured in the BALF recovered from animals killed 18 hours post saline instillation (control), and 18 hours and 72 hours after LPS instillation. There was relatively greater quantity of SP-A in the BALF of animals killed at both 18 and 72 hours after LPS instillation compared to the control animals, consistent with previous reports using similar models of intrapulmonary LPS administration [9-11].

### Whole Lung Studies: Clearance of Radiolabel Liposomes

**Figure 2:**
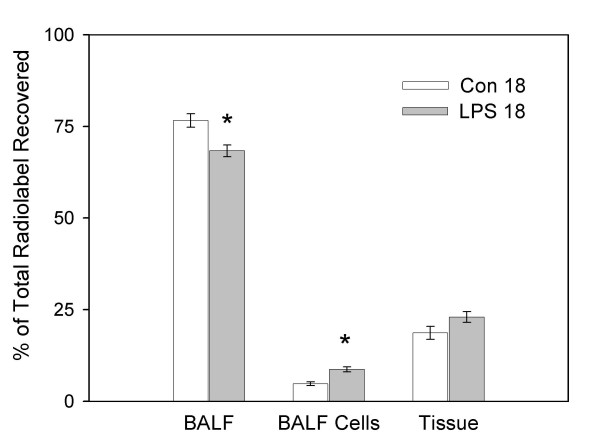
**Distribution of radiolabel liposomes 18 hours after instillation of LPS. **Whole lung distribution of the total recovered ^3^H-liposomes instilled 2 hours prior to killing. Animals were killed 18 hours after instillation of saline (Con 18) or 100 μg/kg O26:B6 lipopolysaccharide (LPS 18) where the lungs were lavaged, lavage cells isolated and whole lung tissue homogenized. Radioactivity was subsequently measured in cell free bronchoalveolar lavage fluid (BALF), isolated BALF cells and whole lung tissue homogenate (Tissue). Data are means ± SEM and expressed as a percentage of total recovered radiolabel; n = 6 animals/group. Statistical significance * = p < 0.05 vs. respective control group.

Figure 2 shows distribution of the recovered radioactivity associated with the cell free BALF, the isolated BALF cells, and whole lung tissue for the two groups killed 18 hours after instillation of saline or LPS. There was no difference in total lung radioactivity recovered compared to the total radiolabel instilled between the Control 18 and LPS 18 groups (46.0 ± 4.6% and 48.4 ± 4.0%; respectively). There was significantly less radiolabel recovered in the BALF (p < 0.05) and significantly more radiolabel associated with the BALF cells (p < 0.01) in the LPS group compared to the control group. There was no difference in radiolabel association with lung tissue between the two groups.

**Figure 3:**
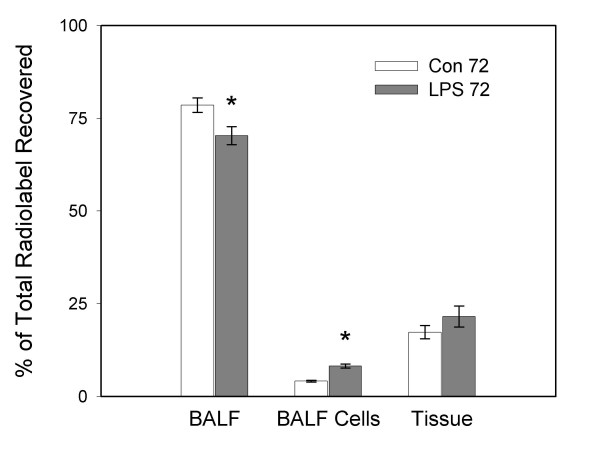
**Distribution of radiolabel liposomes 72 hours after instillation of LPS. **Whole lung distribution of the total recovered ^3^H-liposomes instilled 2 hours prior to killing. Animals were killed 72 hours after instillation of saline (Con 72) or 100 μg/kg O26:B6 lipopolysaccharide (LPS 72) where the lungs were lavaged, lavage cells isolated and whole lung tissue homogenized. Radioactivity was subsequently measured in cell free bronchoalveolar lavage fluid (BALF), isolated BALF cells and whole lung tissue homogenate (Tissue). Data are means ± SEM and expressed as a percentage of total recovered radiolabel; n = 6 animals/group. Statistical significance * = p < 0.05 vs. respective control group.

Figure 3 displays total recovered radioactivity associated with the cell free BALF, the isolated BALF cells, and whole lung tissue for the two groups killed 72 hours after instillation of saline or LPS. There was no difference in the total lung radiolabel recovered compared to the total radiolabel instilled between the Control 72 and LPS 72 groups (47.3 ± 4.0% and 44.8 ± 3.8%; respectively). There was significantly less radiolabel recovered in the BALF (p < 0.05) and significantly more radiolabel liposomes associated with the BALF cells (p < 0.01) in the LPS group compared to the control group. There was no difference in radiolabel associated with lung tissue between the two groups.

Additional controls were performed in which animals (n = 2) received radiolabel liposomes followed by whole lung lavage and lung tissue homogenization 5 minutes after the instillation procedure. Mean total lung radiolabel recovery for this control group was 77% suggesting that ~25% of the radiolabel liposomes were lost in the instillation procedure (i.e., syringe, catheter), or possibly were adhered to the airway epithelium. The distribution of the recovered liposomal radioactivity within the 5-minute control lungs was 91% associated with the BALF, 2% associated with BALF cells, and 7% associated with lung tissue.

### Type II Cell Isolation: Cell Recoveries, Purities and Radioactivity per Cell

Table 2 demonstrates the cell recovery and cell differential after type II cell isolation for the two experimental groups at the 18-hour time point. Type II cell recovery for the control group ranged from 10.4 × 10^6 ^to 21.6 × 10^6 ^cells with a mean of 17.1 × 10^6^. The purity of this cell fraction averaged greater than 80% type II cells and viability was greater than 95%. Plate cell recovery from the control group ranged from 1.8 × 10^6 ^to 4.7 × 10^6 ^cells with a mean value of 3.2 × 10^6^. This cell fraction was predominately macrophages and the viability was greater than 90%. For the LPS group at the 18-hour time point, type II cell recovery ranged from 10.6 × 10^6 ^to 20.5 × 10^6 ^cells and a mean of 16.3 × 10^6 ^cells, with a purity greater than 85% type II cells and viability greater than 95%. Plate cells from the LPS 18 group ranged from 2.7 × 10^6 ^to 13 × 10^6 ^cells with a mean of 6.6 × 10^6 ^cells. This cell population consisted primarily of neutrophils and macrophages and viability was greater than 90%. There were no significant differences in cell numbers obtained from control and LPS animals 18 hours after saline or LPS instillation.

**Table 2:** Cell recovery and differential after lung digestion and IgG panning for animals killed 18 hours after instillation of saline (Control) or lipopolysaccharide (LPS).

	Control 18 (n = 7)		LPS 18 (n = 7)	
		
	Type II Cells	Plate Cells	Type II Cells	Plate Cells
Cell Numbers (10^6^)	17.1 ± 1.6	3.2 ± 0.5	16.3 ± 1.8	6.6 ± 1.7
% Type II	81 ± 2	15 ± 7	86 ± 2	14 ± 2
% Macrophages	9 ± 1	61 ± 5	3 ± 1*	33 ± 2*
% Neutrophils	1 ± 1	17 ± 10	4 ± 1	53 ± 3*
% Other Cells	9 ± 2	7 ± 2	7 ± 2	-

Values are mean ± SEM. * = p < 0.01 vs. Control.

**Figure 4:**
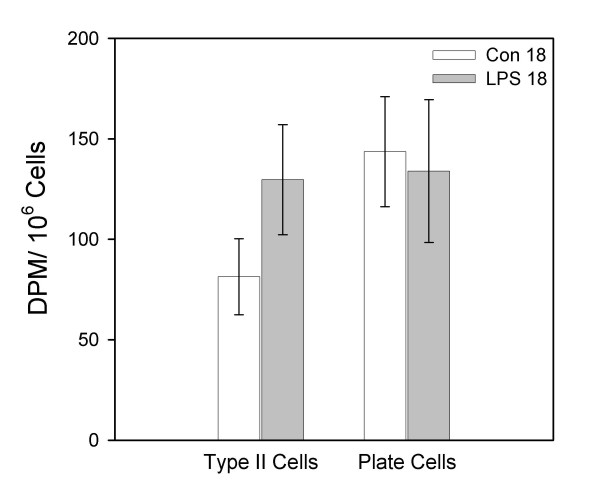
**Liposome uptake by lung tissue cells 18 hours after instillation of LPS. **Association of ^3^H-liposomes instilled 2 hours prior to killing in cells isolated from lung tissue. Animals were killed 18 hours after instillation of saline (Con 18) or 100 μg/kg O26:B6 LPS (LPS 18). Lungs were lavaged and type II cells and other tissue-associated cells (plate cells) were isolated. Data are means ± SEM and expressed as DPM/10^6 ^cells, n = 7 animals/group.

Figure 4 reveals the radioactivity per million tissue cells for the isolated type II cells and the other tissue associated cells (plate cells) for the control and LPS 18-hour groups. There were no significant differences in the radioactivity per type II cell or plate cell between the control group and the LPS group at the 18-hour time point after instillation.

Table 3 shows the cell recovery and cell differential after type II cell isolation for the two experimental groups at the 72-hour time point. Type II cell recovery for the control group ranged from 21.2 × 10^6 ^to 40 × 10^6 ^cells with a mean of 28.4 × 10^6^. The purity of this cell fraction averaged 75% type II cells and viability was greater than 95%. The number of cells obtained from the plate group ranged from 1.8 × 10^6^to 15.9 × 10^6 ^cells with a mean value of 6.2 × 10^6 ^with the primary cell type being macrophages with a small percentage of neutrophils and type II cells. Viability for the plate cells were greater than 90%. For the LPS group at the 72-hour time point, type II cell recovery ranged from 19.2 × 10^6 ^to 64.6 × 10^6 ^cells and a mean of 33.2 × 10^6 ^cells, purity greater than 80% type II cells and viability greater than 95%. Plate cells from the LPS 18-hour group ranged from 3.3 × 10^6 ^to 16.2 × 10^6 ^cells with a mean of 7.8 × 10^6 ^cells. This cell population consisted primarily of macrophages and viability was greater than 90%. There were no significant differences in cell recoveries between the control and LPS groups at the 72-hour time point. Of note, there were a greater number of cells recovered from both 72-hour groups compared to both 18-hour groups.

**Table 3:** Cell recovery and differential after lung digestion and IgG panning for animals killed 72 hours after instillation of saline (Control) or lipopolysaccharide (LPS).

	Control 72 (n = 6)		LPS 72 (n = 7)	
		
	Type II Cells	Plate Cells	Type II Cells	Plate Cells
Cell Numbers (10^6^)	28.4 ± 3.9	6.2 ± 2.2	33.2 ± 5.6	7.8 ± 2.2
% Type II Cells	75 ± 2	22 ± 3	83 ± 1	19 ± 3
% Macrophages	11 ± 2	56 ± 5	12 ± 2	59 ± 4
% Neutrophils	-	22 ± 7	2 ± 1	22 ± 3
% Other Cells	14 ± 1	-	3 ± 3*	-

Values are mean ± SEM. * = p < 0.01 vs. Control.

**Figure 5:**
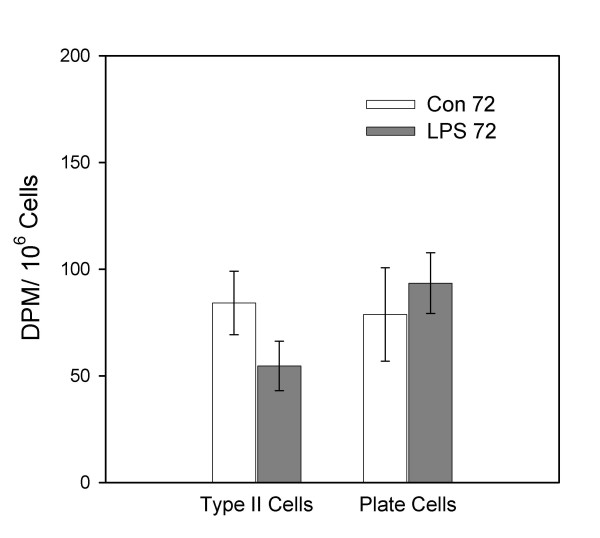
**Liposome uptake by lung tissue cells 72 hours after instillation of LPS. **Association of ^3^H-liposomes instilled 2 hours prior to killing in cells isolated from lung tissue. Animals were killed 72 hours after instillation of saline (Con 72) or 100 μg/kg O26:B6 LPS (LPS 72). Lungs were lavaged and type II cells and other tissue-associated cells (plate cells) were isolated. Data are means ± SEM and expressed as DPM/10^6 ^cells; Con 72 = 6 animals, LPS 72 = 7 animals.

Figure 5 reveals the radioactivity per million tissue cells for the isolated type II cells and the other tissue-associated cells (plate cells) for the control and LPS 72-hour groups. There were no significant differences in the radioactivity per type II cell or plate cell between the LPS group and the control group 72 hours after the appropriate instillation. Of note there was no significant difference in radioactivity per cell for both the type II cells and plate cells between the two 72-hour groups and the two 18-hour groups.

## Discussion

In the present study, we evaluated the clearance of surfactant-like liposomes at 18 and 72 hours following the intrapulmonary instillation of LPS. At both time points, radiolabel liposomes were instilled two hours prior to killing and the distribution within the lung was determined. Radioactivity was measured in cell-free BALF, isolated BALF cells, whole lung tissue, isolated type II cells and remaining tissue-associated cells. At both time points there was a significant increase in clearance of exogenous liposomes from the airspace and a small decrease in alveolar surfactant phospholipid levels in the LPS groups compared to control groups that received vehicle only. This corresponded with increased radiolabel associated with isolated BALF cells and no difference associated with lung tissue at either time point after LPS instillation compared to controls. There was no difference in liposomal radioactivity associated with isolated type II cells or other tissue cells between both LPS groups and their respective control groups, which was in agreement with whole lung measurements. These data suggest that cells recruited into the airspace as a consequence of the LPS-induced inflammatory response can significantly contribute to increased clearance of surfactant-like liposomes from the airspace (Figs. 2 and 3).

Eighteen hours after LPS instillation there was an infiltration of neutrophils into the airspace which resulted in a 10-fold increase in BALF cells. At this time point there was a doubling of total liposomal radioactivity associated with these inflammatory cells compared to alveolar cells recovered from control animals. Neutrophils recruited into alveolar spaces during infection are essential for host defense by phagocytosis and killing of bacteria and other infectious agents. Previous studies have documented that surfactant can in fact modulate neutrophil functions, as surfactant proteins A and D can enhance neutrophil uptake of bacteria [24]. Although these phagocytic cells are extremely important in host defense, they have also been implicated as a contributing factor to the lung injury in a variety of inflammatory disorders. In a recent study by Quintero et al., it was demonstrated by confocal microscopy that neutrophils isolated from inflamed lungs were able to significantly internalize and degrade surfactant lipids *in vitro *[16]. Based on *in vitro *measurements of lipid uptake, they estimated that neutrophils could account for up to 48% of the observed clearance and could significantly impact surfactant homeostasis. Data from the current study supports this idea by demonstrating that *in vivo*, neutrophils can indeed contribute to the clearance of surfactant lipids during pulmonary inflammation.

At the 72-hour time point after LPS instillation there was an accumulation of macrophages in the alveolar space that resulted in a 5-fold increase in BALF cells compared to control animals. There was a doubling of radiolabeled phospholipids associated with these alveolar cells and no increase in the amount of radiolabeled phospholipids associated with whole lung tissue. This resulted in a significant increased in clearance of total liposomal radioactivity from the BALF, 72 hours after LPS instillation. Alveolar macrophages have been shown *in vitro *to take up and degrade surfactant lipids and have also been shown *in vivo *to account for approximately 20% of surfactant lipid clearance in normal lungs [16,25]. Macrophages isolated from LPS-exposed lungs were shown to have a greater capacity to take up surfactant lipids *in vitro *than macrophages isolated from normal lungs [16], suggesting that in an inflamed lung, the recruited macrophages may have a significant role in surfactant metabolism. Indeed, the *in vivo *data presented in the current study support the theory that activated alveolar macrophages can impact clearance of surfactant lipids.

Of note, previous *in vitro *data had predicted that inflammatory cells (neutrophils and macrophages) could account for a 6 to 13 fold increase in lipid clearance [16]. In the current study there was a 5–10 fold increase in alveolar inflammatory cells but only a doubling of cell associated radioactivity and a change in alveolar phospholipid pool size of approximately 15%. Importantly, the current study instilled a similar dose of LPS, had similar end points and the same number of inflammatory cells recovered from the BALF as the aforementioned *in vitro *study. Although the present *in vivo *data demonstrated that the inflammatory cells can indeed contribute to increased lipid clearance as suggested previously, the absolute value was considerably less than that predicted from the *in vitro *studies. Although we do not have an explanation for this quantitative difference between the *in vivo *and *in vitro *observations, it is possible that the process of cell isolation in the *in vitro *study resulted in their activation for subsequent lipid uptake. Alternatively, *in vivo*, multiple factors can impact total surfactant pool size in addition to clearance by inflammatory cells.

Type II cells are the predominant cell type that regulates surfactant metabolism, being involved in synthesis, secretion and clearance. Secretion of surfactant phospholipids is solely a property of alveolar type II cells, whereas clearance in a healthy lung is regulated primarily by both type II cells and alveolar macrophages. In one study utilizing uninjured rabbits, it was determined that type II cells accounted for approximately 65% of the clearance of alveolar phospholipid [25]. In a situation of pulmonary inflammation induced by LPS administration, there have been documented decreases in alveolar surfactant phospholipids [9-11], increases in intracellular surfactant phospholipids [10] and the appearance of giant lamellar bodies within type II cells [17,18,26]. From these observations, LPS induced inflammation could either inhibit surfactant secretion or enhance surfactant clearance by type II cells, resulting in diminished alveolar surfactant pool and increased intracellular pool. We originally hypothesized that after LPS administration there was increased clearance of surfactant phospholipids by type II cells. However, data from this study demonstrated that *in vivo*, surfactant clearance by type II cells was similar at both 18 and 72 hours after intrapulmonary LPS instillation compared to control groups killed at the same time points (Figs 4 and 5). Data from the current study cannot lead to any conclusions pertaining to alterations in type II cell phospholipid synthesis or secretion.

As mentioned, we observed a small but not statistically significant decrease in alveolar phospholipid pools after LPS administration, which is in contrast to previous studies by Viviano et al. and MacIntosh et al. that used 1 mg/kg and 0.5 mg/kg of LPS respectively [9,10]. Nevertheless, we did observe a similar increase in alveolar levels of SP-A after intrapulmonary LPS (Fig 1), which is a well documented characteristic of lung inflammation after LPS exposure [9-11]. This increase has been primarily attributed to increased SP-A synthesis by type II cells [11] suggesting that indeed type II cell metabolism was altered in our model. Additionally, these data provide further experimental evidence that surfactant phospholipid and SP-A pools can be independently regulated in response to inflammatory agents.

We also investigated additional tissue-associated cells that were isolated after lung digestion (primarily macrophages and neutrophils). Similar to type II cells, surfactant clearance from these tissue-associated cells isolated from the two LPS groups were similar to their respective control groups. In addition, the clearance attributed to type II cells and tissue-associated cells was similar at both time points for the LPS and control groups. This observation is in agreement with a study by Gurel et al., that documented that alveolar type II cells and tissue macrophages contributed equally to the alveolar clearance of phospholipid in adult mice [27].

## Conclusions

In conclusion, we demonstrated that after intrapulmonary LPS administration in adult rats, the *in vivo *clearance of surfactant lipids by type II cells is unaltered and that the increased clearance of surfactant lipids is primarily due to either neutrophils or macrophages that are recruited to the alveolar space.

## Authors' contributions

JLM conceived, designed and performed all aspects of the study, and was the primary participant in its writing. JRW aided in conception and design of study, and participated in its writing.
